# ChemDIS: a chemical–disease inference system based on chemical–protein interactions

**DOI:** 10.1186/s13321-015-0077-3

**Published:** 2015-06-15

**Authors:** Chun-Wei Tung

**Affiliations:** School of Pharmacy, College of Pharmacy, Kaohsiung Medical University, 100 Shih-Chuan 1st Road, Kaohsiung, 80708 Taiwan; Ph.D. Program in Toxicology, College of Pharmacy, Kaohsiung Medical University, Kaohsiung, 80708 Taiwan; National Environmental Health Research Center, National Health Research Institutes, Miaoli County, 35053 Taiwan

**Keywords:** Chemical–disease inference, Chemical–protein interaction, Gene ontology, Disease ontology, Enrichment analysis

## Abstract

**Background:**

The characterization of toxicities associated with environmental and industrial chemicals is required for risk assessment. However, we lack the toxicological data for a large portion of chemicals due to the high cost of experiments for a huge number of chemicals. The development of computational methods for identifying potential risks associated with chemicals is desirable for generating testable hypothesis to accelerate the hazard identification process.

**Results:**

A chemical–disease inference system named ChemDIS was developed to facilitate hazard identification for chemicals. The chemical–protein interactions from a large database STITCH and protein–disease relationship from disease ontology and disease ontology lite were utilized for chemical–protein–disease inferences. Tools with user-friendly interfaces for enrichment analysis of functions, pathways and diseases were implemented and integrated into ChemDIS. An analysis on maleic acid and sibutramine showed that ChemDIS could be a useful tool for the identification of potential functions, pathways and diseases affected by poorly characterized chemicals.

**Conclusions:**

ChemDIS is an integrated chemical–disease inference system for poorly characterized chemicals with potentially affected functions and pathways for experimental validation. ChemDIS server is freely accessible at http://cwtung.kmu.edu.tw/chemdis.

**Electronic supplementary material:**

The online version of this article (doi:10.1186/s13321-015-0077-3) contains supplementary material, which is available to authorized users.

## Background

Humans are exposed to thousands of chemicals in everyday life. Nevertheless, the toxicological data required for risk assessment are largely unknown for a large portion of chemicals. Instead of applying in vitro or in vivo experiments directly that are expensive and time-consuming, the computational integration of existing toxicogenomics information for the inference of potential toxicities and pathways could largely accelerate the process of risk assessment.

For the integration of toxicogenomics information, the Comparative Toxicogenomics Database (CTD) was constructed by curating chemical–gene/protein interactions from more than 100,000 selected articles for a decade [1, 2]. The chemical–gene–disease associations could be inferred by combining chemical–gene interactions with gene–diseases associations. CTD consisting of high-confidence chemical–gene interactions is a useful resource for studying chemical-induced diseases. Please note that the inferred associations could be either therapeutic or toxic effects. While the analysis of chemical–gene/protein interactions could be useful for narrowing down potentially affected diseases, the interactions alone can not be used to determine whether a chemical induces therapeutic or toxic effects due to the complex nature of biological systems involving various interaction types. Experiments should be subsequently applied to determine which effects are associated with a given chemical. In spite of the limitation, the inference analysis is capable of identifying a small subset of potentially affected diseases with interacting genes/proteins for experimental validation that greatly accelerates the hazard identification process. Traditional bioassays are usually designed for a few specific toxicological and pharmacological endpoints. The integrated analysis of interactions reported from individual toxicology and pharmacology studies is of great importance giving systematic effects that may not be easily observed from the individual studies. However, for poorly characterized chemicals, only a few interacting genes were curated in CTD making the inference of potential diseases impossible.

Instead of analysis of enriched diseases from all interacting genes, ChemProt [3] and HExpoChem [4] focused on analyzing diseases for each chemical-interacting gene/protein based on protein–protein interactions. Although the one-by-one analysis of diseases for each gene could be helpful for studying chemical-induced diseases, a systematic enrichment analysis based on all interacting genes/proteins could provide overall effects that are more easily interpretable.

Recently, a computational inference approach was proposed to identify potential diseases associated with maleic acid, a poorly characterized chemical with only one gene curated in CTD database [5]. The utilization of chemical–protein interaction data from STITCH 3.1, one of the largest chemical–protein interaction databases [6], enabled the inferences of functions, pathways and diseases affected by maleic acid. The approach is potentially useful for the identification of diseases associated with poorly characterized chemicals.

In order to facilitate the inferences of functions, pathways and diseases affected by various environmental and industrial chemicals, a comprehensive resource named ChemDIS was constructed by integrating the chemical–protein interactions in human from STITCH database and enrichment analysis tools. The newly published STITCH 4 with 45% more high-confidence interactions than its previous version [7] was integrated that enlarged the applicability domain of ChemDIS to poorly characterized chemicals. Tools for the enrichment analysis of gene ontology (GO) terms [8], pathways (KEGG [9] and Reactome [10]), disease ontology (DO) [11] and disease ontology lite (DOLite) [12] were implemented and integrated in ChemDIS.

The usefulness of ChemDIS for poorly characterized chemicals was demonstrated by an analysis of maleic acid and sibutramine. ChemDIS successfully inferred kidney diseases that were reported in a safety assessment of maleic acid [13] but not identified in our previous study [5]. In addition, newly identified immune system and infectious diseases provide directions for future studies. For the analysis of sibutramine, the previously reported adverse effects including hypertension, myocardial infarction, heart disease, anorexia nervosa and bipolar disorder [14–17] were also successfully identified by ChemDIS. ChemDIS with user-friendly interfaces is expected to be a useful server for identifying potential risks associated with poorly characterized chemicals.

## Implementation

ChemDIS was constructed by integrating chemical–protein interaction data from STITCH database with various enrichment analysis tools for chemical–disease inference. The analysis functions were implemented using R and Rserv. User interfaces were implemented using HTML, PHP, JavaScript, JQuery and Yadcf (Yet Another DataTables Column Filter [18]). Autocomplete function for chemical search was implemented based on JQuery and Redis, an advanced key-value cache and store database [19].

**Figure 1:**
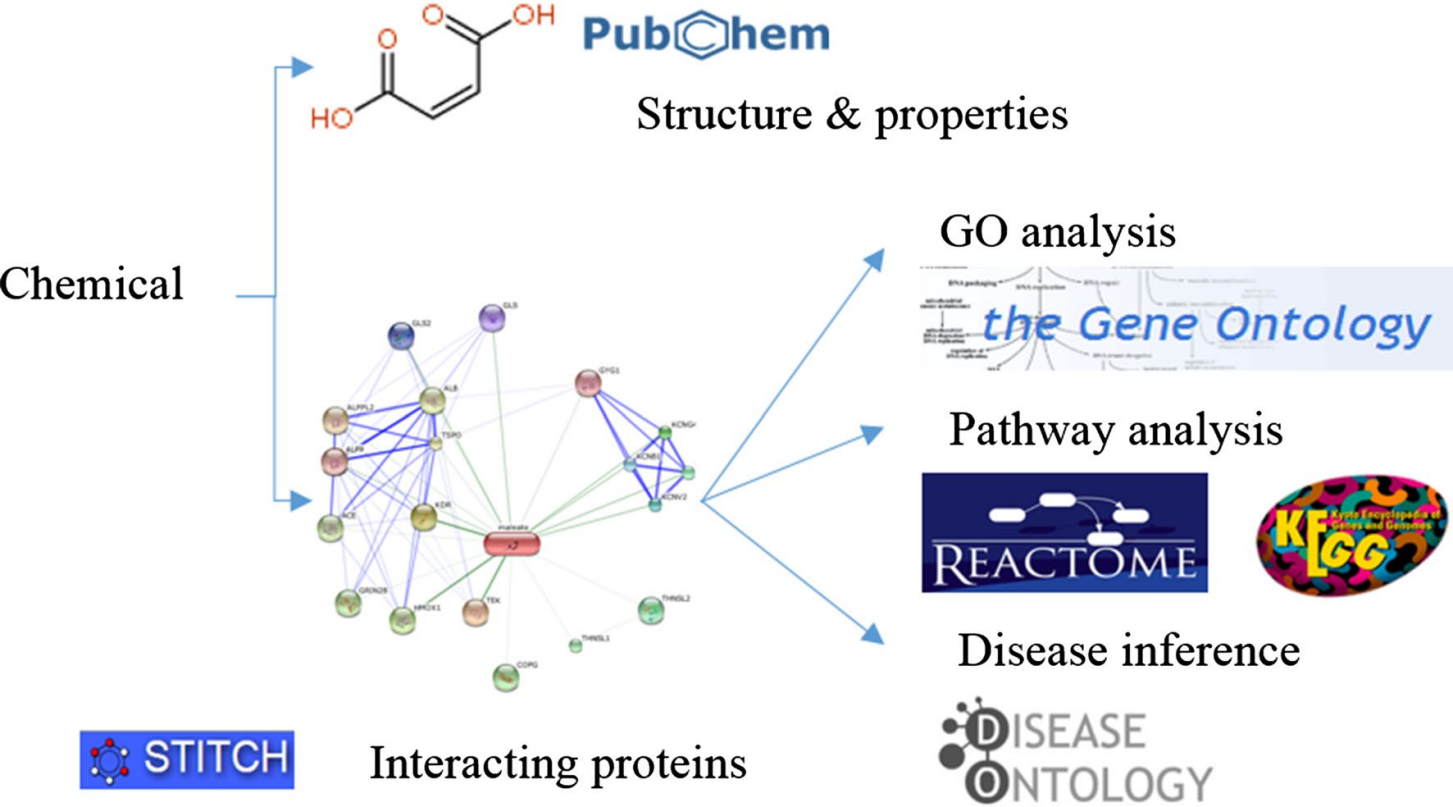
System flow of ChemDIS system.

Figure 1 shows the system flow of ChemDIS. The chemical information of structures and physicochemical properties was downloaded from PubChem [20]. OpenBabel [21] was applied to represent the 2D structures of chemicals. Chemical–protein interaction data were retrieved from STITCH database, an aggregated database of interactions from several interaction databases [7], such as CTD [5], ChEMBL [22], DrugBank [23], Kyoto Encyclopedia of Genes and Genomes (KEGG) [9] and Reactome [10]. The aggregated interaction data were also shown to be useful for predicting non-genotoxic hepatocarcinogenicity [24]. Both STITCH databases of versions 4 and 3.1 were integrated into ChemDIS connecting over 300,000 chemicals and 19,489 and 16,973 human proteins, respectively. The combined scores obtained from STITCH were used for filtering interacting proteins with three confidence levels of high, medium and low.

Enrichment analysis tools were implemented and integrated into ChemDIS for analyzing functions, pathways and diseases affected by a given chemical. For enriched functions, clusterProfiler [25] will be applied to analyze enriched gene ontology (GO) terms for molecular function, biological process and cellular component. The enriched KEGG [9] and Reactome [10] pathways will be analyzed using clusterProfiler and ReactomePA [26], respectively. For inferring diseases affected by a given chemical, enriched DO [11] and DOLite [12] terms will be analyzed using DOSE package [27]. DOLite is a simplified vocabulary list from DO, a standardized ontology connecting human proteins to diseases. All the enrichment analyses are based on hypergeometric tests with the Benjamini–Hochberg approach [28] for multiple testing correction. Enriched terms with a corrected *p* value <0.05 will be identified.

## Results and discussion

### ChemDIS

**Figure 2:**
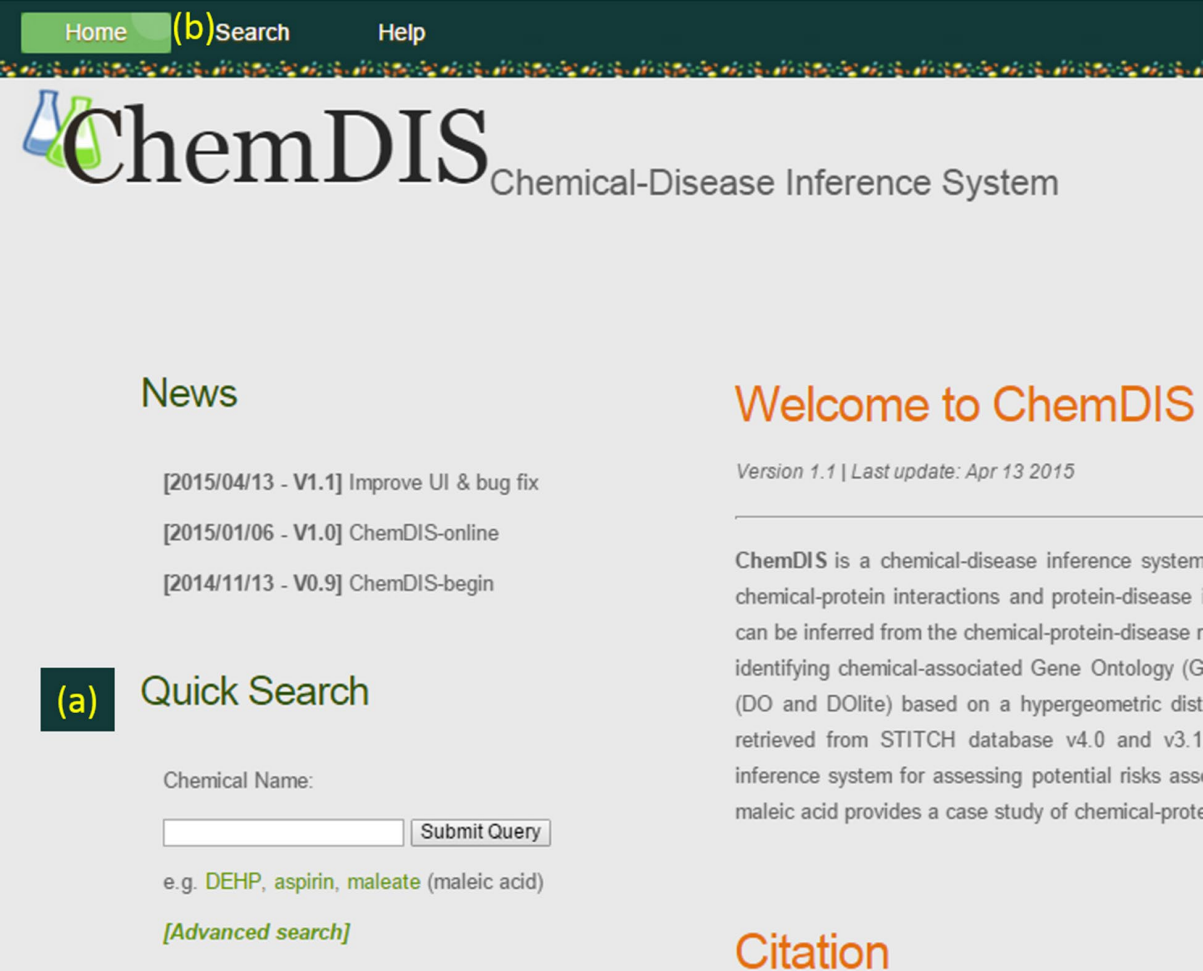
The user interface of ChemDIS providing two search tools: **a** the quick search and **b** advanced search.

ChemDIS provides a unique resource for inferring functions, pathways and diseases associated with chemicals based on chemical–protein interactions. Figure 2 shows the web-interface of ChemDIS equipped with a quick search tool and a hyperlink to advanced search tool. The quick search tool utilizes default parameters of 0.15 for interaction score and 4 for database version. Given a chemical queried by users, its basic structure and property information including chemical 2D structure, hydrogen-bond acceptor, hydrogen-bond donor, IUPAC name, INCHI, INCHIKEY, molecular formula, molecular weight, canonical SMILES, isomeric SMILES and topological polar surface area (TPSA) is available at ChemDIS. To give insights into the functions and pathways affected by chemicals, built-in functions are available for analyzing enriched GO terms and pathways (KEGG and Reactome).

Diseases associated with chemicals will be inferred from their interacting proteins based on DO and DOLite. The utilization of standardized DO terms integrated from multiple ontology sources [11] is expected to provide comprehensive analysis results, while DOLite terms offer simplified disease terms that are more interpretable. Hyperlinks to external databases are available for detailed information of chemicals, proteins, genes, GO, pathways and DO. Result tables are sortable by clicking the header of tables with search functions for filtering results. All analysis results generated from ChemDIS are downloadable.

Due to the dependence of ChemDIS on chemical–protein interactions, the number of interactions for a given chemical determines its applicability domain. For chemicals with more than or equal to 30 interacting human proteins, 14,831 chemicals can be analyzed at ChemDIS, compared with 1,190 chemicals and 2,097 chemicals for CTD without and with the incorporation of cross-species interactions, respectively (Access date: April 5, 2015). Table 1 shows the detailed comparison of ChemDIS and CTD. ChemDIS utilizing chemical–protein interactions from the large database STITCH 4 enables the inference of potential risks for a wide range of chemicals.

**Table 1:** Comparison of ChemDIS and CTD

	ChemDIS	CTD (Apr 5, 2015)
Source of interactions for disease inference	STITCH	Manual curation
No. of chemical–gene/protein interactions	4,523,609 (human)	397,051 (human) 1,041,256 (all species)
No. of chemicals (≥1 interacting genes/proteins)	96,218 (human)	7,432 (human) 10,837 (all species)
No. of chemicals (≥30 interacting genes/proteins)	14,831 (human)	1,190 (human) 2,097 (all species)
GO analysis	GO	GO
Pathway analysis	Reactome and KEGG	Reactome and KEGG
Disease analysis	DO and DOLite	MEDIC
No. of disease terms	8,727 (DO) 561 (DOLite)	11,885

### Case study of maleic acid

As a case study, the potential risks of maleic acid on human health were reanalyzed using ChemDIS and compared with our previous study [5]. Based on STITCH 4, 36 genes mapped from maleic acid-interacting proteins were identified using the keyword ‘maleate’, a synonym of maleic acid, and default threshold 0.15 for interaction score that all interacting proteins will be utilized for the following analysis. Hyperlinks to Ensembl [29] protein database and NCBI Gene database were also available for detailed information.

Both neuronal system and metabolism were identified to be potentially affected by maleic acid from GO and pathway enrichment analyses that were consistent with our previous report. Hyperlinks to external databases of QuickGO [30, 31], KEGG and Reactome were also available at ChemDIS. DO enrichment analysis confirmed that disease of mental health, nervous system disease and disease of metabolism could be potentially associated with maleic acid. The identification of cardiovascular diseases was also consistent with our previous study.

**Figure 3:**
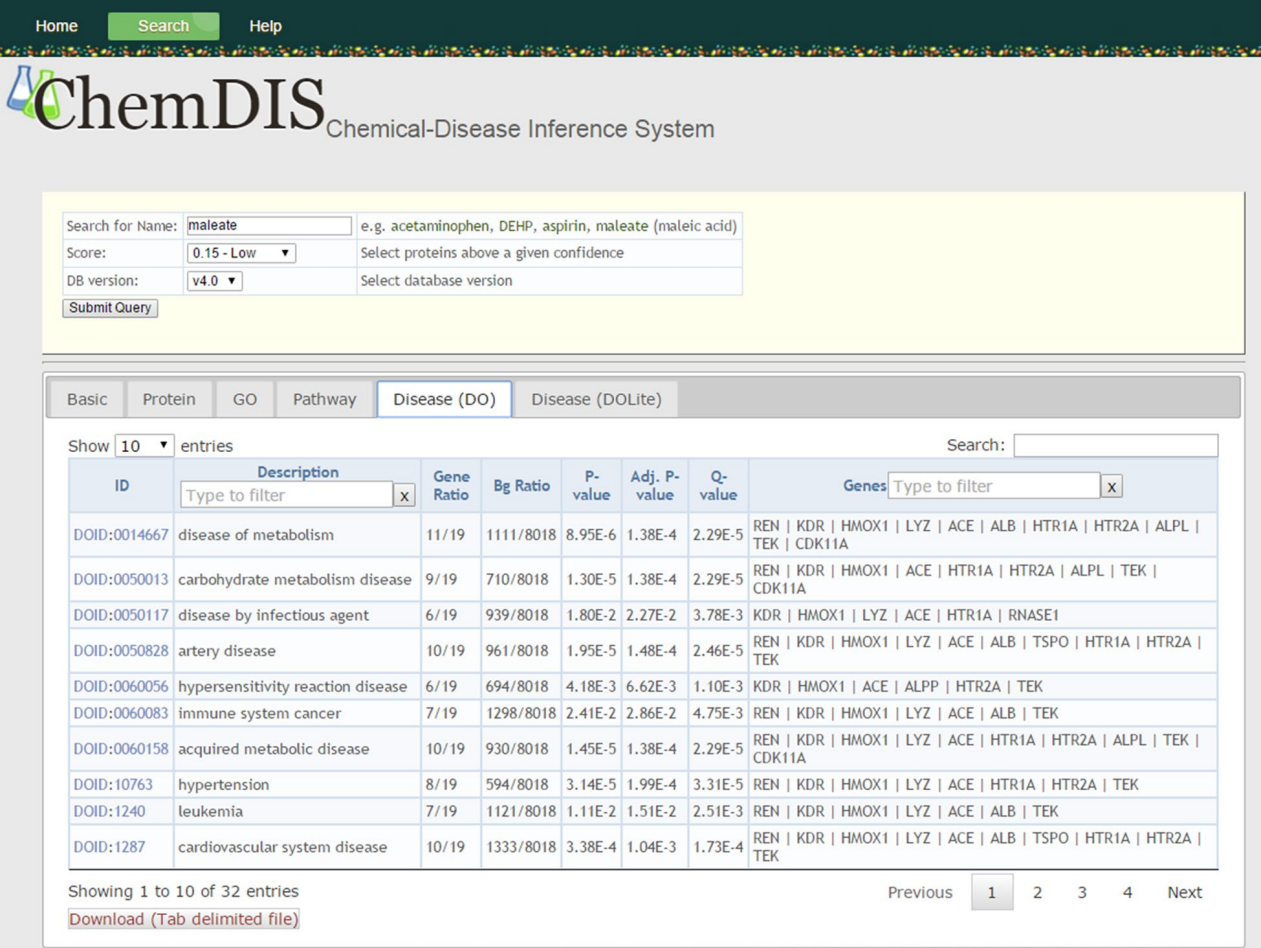
Snapshot of DO term enrichment analysis for maleic acid.

A snapshot of DO enrichment analysis for maleic acid is shown in Figure 3. The gene ratio indicates the ratio between the number of interacting genes associated with a DO term and the number of interacting genes mapped to DO terms. The ratio between the number of genes associated with a DO term and the number of genes mapped to DO terms is represented as background ratio (Bg Ratio). The *p* value and adjusted *p* value are calculated based on the hypergeometric test without and with multiple test correction, respectively. The *q* value is a measure of false discovery rate [32].

Newly identified diseases included immune system, kidney and infectious diseases. Notably, kidney diseases inferred by ChemDIS has been shown in experimental animals that our previous study failed to identify [5]. ChemDIS successfully identified known diseases associated with maleic acid including kidney [13], behavioral and gastrointestinal diseases [33]. DOLite enrichment analysis showed that hypertension could be associated with maleic acid. The detailed analysis results for maleic acid is available in Additional file 1: Table S1. In summary, ChemDIS identified 3 and 29 DO terms for known and newly identified diseases from 8,727 DO terms, respectively. In addition, a newly identified DOLite term of hypertension was identified. The analysis results provide future directions of toxicological research on maleic acid. For CTD, 1 and 56 disease terms were identified for known and newly identified diseases, respectively. Please note that the inference from CTD was based on only one gene giving low-scoring diseases without sufficient information for further experimental validation.

### Case study of sibutramine

In addition to the maleic acid with less known associated diseases, a withdrawal drug sibutramine was used to evaluate the ability of ChemDIS to identify known associated diseases. Similar to maleic acid, there is only five genes curated in CTD database giving only partial interaction information making the disease inference difficult. Sibutramine is originally indicated for the management of obesity and has been withdrawn from the market due to the concern of cardiotoxicity [15, 16]. The reported adverse effects associated with sibutramine include symptoms of cardiovascular, nervous and gastrointestinal system diseases and disease of mental health. Hypertension, myocardial infarction, arrhythmias, tachycardia, stroke, bipolar disorder, headache, insomnia, constipation, anorexia nervosa and sexual dysfunction have been reported to be associated with sibutramine [14–17, 34–36].

ChemDIS identified 44 genes mapped from sibutramine-interacting proteins based on STITCH 4. The DO terms of cardiovascular system disease, hypertension, myocardial infarction and heart disease were successfully identified. The enriched DO term of heart disease accounts the arrhythmias, tachycardia and stroke. ChemDIS performs well for identifying known sibutramine-induced cardiotoxicity. The corresponding DO terms for nervous system disease were also identified including nervous system disease and bipolar disorder. The enriched DO term of nervous system disease implies the symptoms of headache and insomnia. For constipation, the DO term of gastrointestinal system disease was identified. For the disease of mental health, the corresponding DO terms of disease of mental health and anorexia nervosa were identified accounting the adverse effects of sexual dysfunction and anorexia nervosa. A DOLite analysis also successfully identified hypertension and anorexia nervosa. As the interaction data grows, the inferred diseases could be more precise. In addition to the adverse effects, the desired therapeutic effects were also identified as DO terms of obesity, fatty liver disease, overnutrition, nutrition disease and eating disorder and the DOLite term of obesity [37, 38]. The detailed analysis results for sibutramine is available in Additional file 2: Table S2.

Generally, ChemDIS identified 103 DO terms and 7 DOLite terms from a large pool of disease terms that largely help the prioritization of potentially associated diseases. Among the 103 identified DO terms, 10 and 5 terms are consistent with previously reported adverse and therapeutic effects, respectively. For the 7 inferred DOLite terms, there are 2 and 1 terms corresponding to known adverse and therapeutic effects. Newly identified associations include the remaining 88 and 4 terms for DO and DOLite, respectively. For CTD, most of the identified 57 disease terms were low-scoring associations that the average number of genes used for each inference is only 1.12. While 5 and 2 terms from CTD analysis were consistent with the previously reported adverse and therapeutic effects, respectively, it is difficult to experimentally validate the results without sufficient information.

## Conclusions

ChemDIS is an integrated chemical–disease inference system with a user-friendly interface. Benefit from the integration of the large STITCH database, ChemDIS is expected to be helpful for inferring potential diseases associated with poorly characterized chemicals. The integration of analysis tools enabled the identification of affected functions and pathways that can be further studied experimentally. The analysis of maleic acid and sibutramine demonstrated the capability of ChemDIS for identifying a small number of potential affected diseases from the large pool of disease terms. To further improve the applicability of ChemDIS to chemicals without sufficient interaction data, future works could be the implementation of pharmacophore- and docking-based target identification methods such as PharmMapper [39, 40] and PDTD [41], respectively, and incorporation of predicted targets for enrichment analysis.

## Availability and requirements

ChemDIS is freely available at http://cwtung.kmu.edu.tw/chemdis without restrictions for academic use.

## Additional files

Additional file 1: Table S1. Analysis results for maleic acid. Additional file 2: Table S2. Analysis results for sibutramine.
